# Understanding off-target effects through hybridization kinetics and thermodynamics

**DOI:** 10.1007/s10565-019-09505-4

**Published:** 2019-12-10

**Authors:** Nafisa N. Nazipova, Svetlana A. Shabalina

**Affiliations:** 1 Institute of Mathematical Problems of Biology, Keldysh Institute of Applied Mathematics of Russian Academy of Sciences, Pushchino, Moscow Region, Russia; 2 National Center for Biotechnology Information, National Library of Medicine, National Institutes of Health, Bethesda, MD USA

In modern biotechnological and medical research, RNA-guided nucleases (RGNs) continue to be highly effective in targeted modification of genomes and the manipulation of gene expression (Sander and Joung 2014; Wang and Wang 2017). In RNA interference (RNAi) and CRISPR (clustered regularly interspaced short palindromic repeats) -Cas (CRISPR-associated protein) systems, RGNs regulate or modify genes through sequence-specific base-pairing between a short interference or single guide RNAs (siRNAs or sgRNAs) and DNA/RNA targets (Bisaria et al. 2017; Shabalina and Koonin 2008). The patterns of base-pairing interactions may modulate RGN binding affinity and reduce off-targeting (Bisaria et al. 2017; Shabalina et al. 2006). RGNs exhibit off-target behavior when interactions and modifications are made not only in the intended location (on-target) but also elsewhere in the genome where sequences are similar to the intended target (off-target) (Klein et al. 2018; Kempton and Qi, 2019). Nucleotide sequence preferences that improve sgRNA efficiency are substantially different for variable CRISPR-based systems (Kim et al., 2019; Slaymaker et al. 2016; Xu et al. 2015), which is adapted from diverse bacterial defense systems (Koonin et al. 2017; Makarova et al. 2006). Thus, due in part to growing interest in CRISPR-Cas variants, this editorial primarily focuses on off-targeting in CRISPR-Cas systems and comparison with RNAi.

CRISPR-Cas proteins are non-specific endonucleases that bind a protospacer adjacent motif (PAM) located in the proximity of the genomic target (Bollen et al. 2018). sgRNA enables the recognition of the target region of interest through complementary base pairing and directs the Cas nuclease there for specific editing. The sgRNA contains a “seed” region, which is especially responsive to mismatches in duplexes with PAM-proximal nucleotides, but variants or mutations of the target distal to the PAM also modulate off rates (Boyle et al. 2017). The off-target behavior is not surprising due to the divergence of sgRNA targeting systems where different selective pressures result in optimizations of specificities and other important features, such as turnover rates (Kim et al. 2019).

Preliminary screening of potential candidates and prediction of off-target activities were conducted using not only computational and theoretical approaches but also experimental off-target validation (Zhang et al. 2019; Wienert et al. 2019). Improvement in specificity, on-target cleavage activity, and reduction of off-target-cleavage can be achieved through changes in CRISPR-derived nuclease, engineering of sgRNA, and/or Cas-sgRNA delivery modifications. Driving improvements to these parameters is a crucial enabler for RGN-based technologies and the realization of its currently untapped potential (Kim et al. 2019; Bisaria et al. 2017). We will also discuss the importance of thermodynamic and kinetic properties for RGN specificity estimation and reducing off-targeting through optimization of sgRNAs. In addition, we briefly touch on the role of enzyme engineering.

## Selection of efficient target sites

The combination of several features defines RGN off-target effects, including (i) nuclease concentration and features and (ii) target site accessibility, functionality, and uniqueness. The occurrence of complementary sites within the genome, which form highly stable duplexes to guide oligonucleotide, is one of the important determinants of off-target activity. Cas9 and Cas12a (Cpf1) activity can be modulated by chromatin states to varying degrees. Off-targeting is expected to be context dependent because chromatin states and DNA accessibility is tissue/cell- or condition-dependent (Kim et al. 2019). Using the combination of statistical thermodynamics and kinetics, Farasat and Salis (2016) demonstrated that the supercoiling of DNA is an important mechanism tied to the control of Cas9 binding. DNA stretching and bubbles with up to ten mismatches can induce Cas9 off-targeting (Newton et al. 2019). Knockout rates significantly improve when unique target sites are in constitutive upstream exons or in conserved domains/sites with critical gene function. Most of these features were used for computational and theoretical predictions of on-target and off-target activity using different algorithms and web design tools (Zhang et al. 2019; Alkan et al. 2018). Among these methods, data-driven machine learning approaches are very successful at learning rules to model specific datasets, but the models may not be generalizable to new data and systems, or representative of physical or mechanistic relationships (Zhang et al. 2019). Recently, several experimental off-target validation techniques demonstrated successful results, including detection of off-target cleavages throughout the entire genome using approaches, which leveraged advances in next generation sequencing (Wienert et al. 2019).

Empirically derived sets of rules, based primarily on experimental off-targeting data, often include RGN decreased binding affinity to its target (Bisaria et al. 2017), which frequently reduces on-target cleavage. Successful oligonucleotide (sgRNA/siRNA/DNA) targeting is accompanied by an increase in specificity of oligonucleotide, which is defined as the ratio of on-target cleavages to off-targets. In several hybridization experiments and systems, high hybridization specificity and high off-targeting signals (cross-hybridization) are not mutually exclusive (Matveeva et al. 2016, 2018). Notable examples are (i) a model for estimating binding energy of the Cas9-gRNA-DNA complex, based on energy parameters experimentally obtained for relevant interactions between nucleic acids (Alkan et al. 2018), and (ii) a physical framework with rigorous free energy analysis (Zhang et al. 2019) for R-loop formation and sgRNA folding. These models can provide an accurate specificity/efficacy and off-targeting evaluations for sgRNA selections and bridge the gap between experimental structural studies and theoretical predictions. The introduction of nucleic acid duplex energy parameters from experimental measurements (Turner and Mathews 2010) as key components of biophysical models helps to discriminate between highly specific oligonucleotides and improves off-target predictions in different RGN systems, including RNAi (Alkan et al. 2017; Matveeva et al. 2012, 2010, 2007; Shabalina et al. 2006) and CRISPR-Cas systems (Alkan et al. 2018; Farasat and Salis, 2016).

## Determinants and kinetic basis for off-target activities

Despite certain differences in determinants of RNAi and CRISPR-Cas systems, there are important commonalities that could be explained using thermodynamics and hybridization kinetics. In the RNAi studies of mouse Argonaute2 (AGO2) RISC complex formation, several key measurements including association kinetics, equilibrium binding energies, and single turnover cleavage rates allows revealing of important rules for binding and cleavage of targets. Becker and co-authors (Becker et al. 2019) suggested a novel strategy for efficient siRNA design after identifying a specific pattern for guide-target mismatches, which increases the target cleavage rate. These data agree with computational estimations and theoretical predictions of optimal siRNA candidates, where position-dependent patterns of mismatches and thermodynamic features of efficient siRNA candidates exhibited a crucial role (Shabalina et al. 2006). The same strategy using an in vitro high-throughput assay was previously applied to evaluate Cas9 binding effectiveness with target sequences with mutations among nucleotides that bind the sgRNA and PAM (Boyle et al. 2017). Mismatch configurations of specific guide positions led to complex nuclease-dCas9 (dead Cas9) dissociation patterns. Significant variation in association and dissociation was noted and attributed to multiple mismatches between sgRNA and DNA at non-seed bases, implying that Cas9 performance could be influenced by kinetic and thermodynamic adjustment (Boyle et al. 2017).

A comparison of Cas9- and Cas12a-binding experiments showed variable binding kinetics responses to target sequence mutations, which explained why Cas12a enables the selection of DNA sequences more precisely than Cas9 (Strohkendl et al. 2018; Boyle et al. 2017). DNA cleavage of both matched and mismatched targets by CRISPR-Cas12a depends on the rate of DNA target binding. Cas12a tightly binds DNA in two distinct kinetic stages, whereas PAM recognition is followed by a rate-limiting R-loop (a hybrid structure of Cas-RNA and target DNA) propagation. The target DNA of Cas12a extend beyond a seed region and has a specific distinguishing pattern of mismatches across much of the R-loop. These observations support the in vivo DNA cleavage patterns and suggest a late transition state for R-loop formation and readily reversible R-loop propagation. Thus, levels of target specificity as well as off-target effects have significant dynamic range across disparate types of nucleases despite the similarity in formation of R-loops served as sequence-specific binding source. Highly efficient nucleases are likely to show more severe off-target activity (Kim et al. 2019).

For Cas nucleases, targeting rules were empirically established by different groups (Klein et al. 2018): (a) the PAM proximal seed region is extremely sensitive to interference from even single-nucleotide mismatches, while significantly diminished sensitivity to mismatches is characteristic of the distal region (individual Cas pattern); (b) off-targets (outside the seed region) are targeted most strongly when mismatches are dispersed; (c) binding exhibited less sensitivity to mismatches than cleavage; and (d) while still maintaining efficiency, target selectivity can be improved by weakened protein DNA interactions (Klein et al. 2018). These rules have already resulted in improvements in the design and prediction strategies of efficient and specific targets (Kim et al. 2019).

## Hybridization kinetics of intermolecular interactions and off-target activity

Several models and approaches based upon thermodynamics and kinetics have the potential to explain off-targeting patterns for CRISPR-Cas and AGO2, as well as for other systems. To go beyond binding energetics, Klein and co-authors (Klein et al. 2018) kinetically modeled formation of guide-target hybrid using characteristics of transition barriers between metastable states of the hybrid with the nuclease. The study demonstrated that mismatch-pattern dependence and seed region can be attributed to the hybridization kinetics, and that the off-targeting rules (a)–(d) (see above) have a kinetic basis. The model showed that kinetically stalled hybridization produced more promiscuous binding than cleavage. The approach also fared favorably when compared with data from different CRISPR-Cas systems, as well as AGO2, and may be applied to any RGN with significant complementarity between guide and target. The study demonstrated that the specificity of engineered systems can be improved without on-target efficiency reduction.

Bisaria and co-authors (Bisaria et al. 2017) analyzed RGN specificity and off-targeting for RNAi and CRISPR-based genome editing. They considered two kinetic regimes: “rapid equilibrium” and “sticky”. Dissociation of RGN from the target was faster than cleavage in the first regime and was slow in the second. Several studies discussed approaches for shifting between kinetic regimes to the “rapid-equilibrium” state of RNA targeting and presented evidence that RGNs occur in a “sticky” state (Bisaria et al. 2017) that may be valuable for in vivo RNAi and CRISPR systems (Wang et al. 2006). The described kinetic models explain some details of RGN targeting mechanisms and highlight the fundamental similarity between different RGN systems. Another approach for improving of the CRISPR system specificity is related to engineering of RNA hairpin folding onto sgRNA spacer regions (hp-sgRNAs). Spacer secondary structures affect the characteristics of kinetic models including the formation of R-loop and can significantly improve specificity. RNA folding emerged as a key parameter for regulating the activity of CRISPR systems, when applied to five distinct variants of Cas9 and Cas12a (Kocak et al. 2019).

## Perspectives

Attenuating DNA cleavage kinetics can be successfully applied for enhancing gene editing specificity and reducing off-targeting not only to CRISPR systems but also to different engineered nucleases (Becker et al. 2019; Miller et al. 2019). Further understanding of off-targeting mechanisms and basic kinetic features is important for future utility of RGNs, and specifically, in CRISPR-Cas technologies with engineered enzymes as precise genome editing tools. One important future direction is the optimization of multiplexed genome engineering approaches with the possibility of simultaneous modification of multiple genetic elements, which are specifically located in non-coding genome regions (Campa et al. 2019; Reis et al. 2019). Another crucial future prospect is the creation of a platform to characterize kinetic and thermodynamic properties of the growing variety of CRISPR nucleases. Comparison of CRISPR, RNAi, and other RGN systems and analysis of their universal features and descriptive parameters can generate mutually beneficial knowledge and cross-talk between these systems.
